# Docking, thermodynamics and molecular dynamics (MD) studies of a non-canonical protease inhibitor, MP-4, from *Mucuna pruriens*

**DOI:** 10.1038/s41598-017-18733-9

**Published:** 2018-01-12

**Authors:** Ashish Kumar, Harmeet Kaur, Abha Jain, Deepak T. Nair, Dinakar M. Salunke

**Affiliations:** 1 Regional Centre for Biotechnology, NCR Biotech Science Cluster, 3rd Milestone, Faridabad-Gurgaon Expressway, Faridabad, 121001 India; 2 Manipal University, Manipal, 576104 Karnataka India; 3 International Centre for Genetic Engineering and Biotechnology (ICGEB), Aruna Asaf Ali Marg, New Delhi, 110067 India; 4 Present Address: The Pennsylvania State University, 469 North Frear, University Park, PA 16802 USA

## Abstract

Sequence and structural homology suggests that MP-4 protein from *Mucuna pruriens* belongs to Kunitz-type protease inhibitor family. However, biochemical assays showed that this protein is a poor inhibitor of trypsin. To understand the basis of observed poor inhibition, thermodynamics and molecular dynamics (MD) simulation studies on binding of MP-4 to trypsin were carried out. Molecular dynamics simulations revealed that temperature influences the spectrum of conformations adopted by the loop regions in the MP-4 structure. At an optimal temperature, MP-4 achieves maximal binding while above and below the optimum temperature, its functional activity is hampered due to unfavourable flexibility and relative rigidity, respectively. The low activity at normal temperature is due to the widening of the conformational spectrum of the Reactive Site Loop (RSL) that reduces the probability of formation of stabilizing contacts with trypsin. The unique sequence of the RSL enhances flexibility at ambient temperature and thus reduces its ability to inhibit trypsin. This study shows that temperature influences the function of a protein through modulation in the structure of functional domain of the protein. Modulation of function through appearance of new sequences that are more sensitive to temperature may be a general strategy for evolution of new proteins.

## Introduction

The recognition of target molecules by protein receptors is critical for many physiological events. The elucidation of protein behaviour in the context of interactions with the corresponding receptor remains a challenging task. Proteins generally have a core stable structure and loop regions that may exhibit flexibility and are deeply associated with function. Over decades, many kinetics and thermodynamics analyses have been performed on proteins to evaluate the function/s corresponding to the dominant structure. The functional state of a protein molecule is a subset of its folding free energy landscape defined by its dynamics. The free energy of a molecule is a function of its enthalpic and entropic contributions. The microenvironment and physico-chemical factors impose conditional topological constraints leading to formation of different microscopic states. Temperature is one such important and crucial factor governing flexibility of macromolecules. Hence a molecule will assume different conformational states as a function of temperature. Of these states, functionally relevant could be one which is optimal. The functionally relevant conformation of a macromolecule aids in interaction with other biological molecules such as proteins, nucleic acids, ligands etc. to carry out diverse physiological functions^1–4^.

One such well-known example is protease and protease inhibitor that ubiquitously span the evolutionary tree from microorganisms to plants to animals. These macromolecules show an enduring evolutionary divergence of their interacting molecules for performing various physiological functions in efficiently regulated manner. Analysis of these macromolecules hints that the functional diversity could be achieved either through change in critical residues participating in interaction or change in the prototypical fold. This enables protease inhibitors to regulate broad-spectrum biological process by controlling the specific endogenous enzymes^5,6^. It has been observed that modulation of protease activity is achieved through blocking, altering or preventing the access of enzyme’s catalytic site^7,8^. These inhibitors follow conventional ways to inhibit the proteases known as the standard or canonical or Laskowski mechanism also well known as the lock-and-key mechanism^9^. In majority of the cases, it has been seen that once docked at the catalytic site, the scissile bond of protease inhibitor is not completely hydrolysed by the nucleophilic attack of amino acid and hence remains occupied at the catalytic site^10,11^. Binding can also influence flexibility of other regions of the protein that are the foundation of allostery and cooperativity^12–14^. Therefore, flexibility in the molecule can be massive, leading to drastic change in the framework of the molecule and can be subtle and restricted to few residues of the protein. Reports indicate that flexibility is not uniformly distributed phenomenon in macromolecules although it has been observed that the regions with high flexibility are more prone to interactions as well as to mutations^15,16^. For protease inhibitors, it has been observed that the surface is covered by flexible loop in majority of the cases but not every loop has the potential to inhibit proteases. Prime reason behind this is geometrical adaptability required for optimal function.

As aforementioned, temperature is an indispensable factor governing catalytic activity. Its role has been elucidated in enzyme-substrate reactions. The dynamics of inhibitory and enzymatic functions of proteins is timescale dependent and is modulated by temperature. The timescale can be short (nano or femtoseconds) or long (minutes to hours or more) depending on the magnitude of topological alteration required for a specific function. The induced or environmental thermal fluctuations change the framework of proteins, thereby altering the potential interactions between the molecules. These changes ultimately affect the magnitude of physiological functions. These changes can be very well monitored through thermodynamics parameters (enthalpy, entropy, and spontaneity). The effect of any unfavourable physiological factors such as temperature, pressure, concentration etc. can be well reflected in thermodynamics parameters. Therefore, a detailed thermodynamic analysis can provide better insights about the forces governing recognition and interaction. Since thermodynamics and kinetics studies on protease inhibitors is sparse and for a complete understanding of structure-function behaviour, it is imperative to investigate the same. So, we chose a protease inhibitor that has not been studied earlier and wanted to investigate temperature dependent change in its structural dynamics. Structural analysis of such complexes can enable estimation of changes in time-dependent structural parameters such as alteration in the B-factors of localized or interacting residues at the binding sites. This will help gain insights on functional activity of these macromolecules^17^.

This paper analyses the relatively weak binding of the protease inhibitor homologue, MP-4, from *Mucuna pruriens*^18^ to the protease trypsin as a function of temperature. The protein, MP-4, is stable in a variety of conditions. Through rationally designed biochemical, thermodynamics and molecular dynamics simulation experiments, the effect of temperature on trypsin binding is analysed. The results suggest that temperature plays an important role in the optimal performance of the complex. The RSL in MP-4 exhibits high conformational flexibility even at ambient temperature. The observations made during the course of the study suggests that at low temperature, the molecule is in a frozen state and relatively rigid for effective interactions. On the other hand, higher temperature amplifies thermal mobility of the molecule that adversely affects its stable binding to respective ligand. However, at an optimal temperature, the functionally important loop is endowed with appropriate adaptability to effectively bind and inhibit the target protease. Overall, it is possible that the unique sequence of the RSL results in heightened conformational flexibility and consequent loss of inhibitory activity. Mutations that lead to modulation of temperature-dependent mobility of functional regions may therefore result in the evolution of new proteins that can be selected for novel functions.

## Results

### Effect of physicochemical parameters on stability of purified MP-4

**Figure 1:**
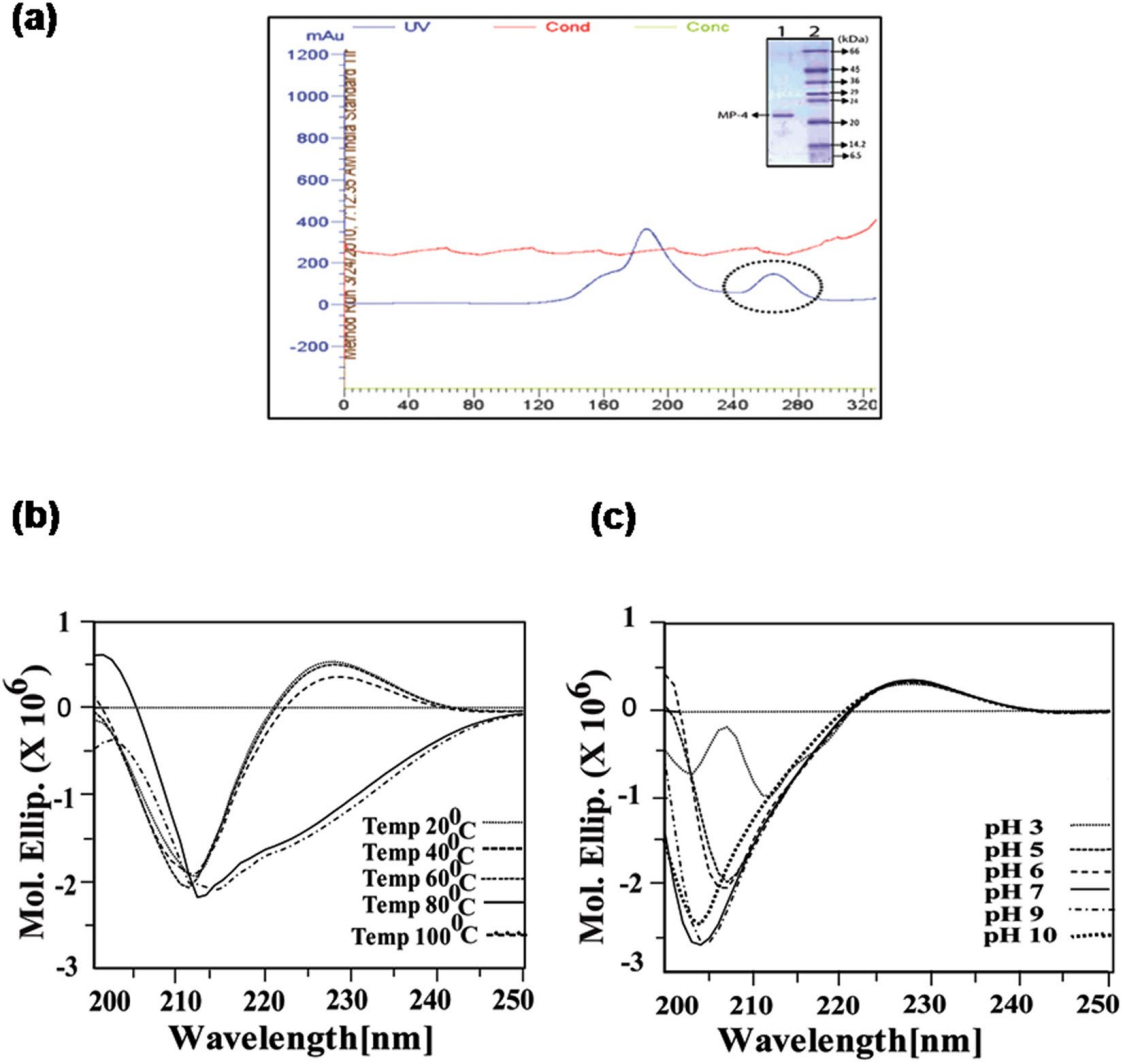
MP-4 purification and stability analysis through circular dichroism (CD). Purification, thermal, and pH stability of MP-4 **(a)** Size exclusion chromatography profile depicting first two peaks which are MP2/MP3 and third peak (circled) is of MP-4. Inset is 12% SDS PAGE indicating the single band of MP-4 protein. **(b)** Effect of various temperatures (20 °C to 100 °C). **(c)** Stability of MP-4 at the range of pH 3–10.

MP-4 was found to be a dominant protein in *Mucuna pruriens* seed proteome and was purified from 60% ammonium sulfate fractionation using gel filtration chromatography (Sephacryl-200 column) (Fig. 1a). Further, purity of MP-4 was ensured through analysis of 12% SDS-PAGE (Fig. 1a inset). This monomeric protein was further analysed for its stability.

The effect of temperature and pH on MP-4 was analysed through circular dichroism (CD) spectroscopy. Data showed that MP-4 is stable from 20 °C to 60 °C as no significant changes in spectra were observed. While beyond 60 °C minor change was seen in the spectrum (Fig. 1b). The spectral peak started shifting towards 220 nm wavelength, indicating the initiation of unfolding of the protein.

Various suitable buffers were used in order to check the effect of pH on stability of MP-4. Even at high pH (pH 10), no significant change in molar ellipticity values were observed (Fig. 1c). On the contrary, a marked change in the spectrum was seen at pH 3. This implies that the structural transition had started between pH 5 to pH 3. Interestingly, the protein precipitated on lowering the ionic strength. These analyses indicated that MP-4 is stable across a wide range of temperature and pH conditions.

### Kinetics and thermodynamics of MP-4 trypsin binding

**Figure 2:**
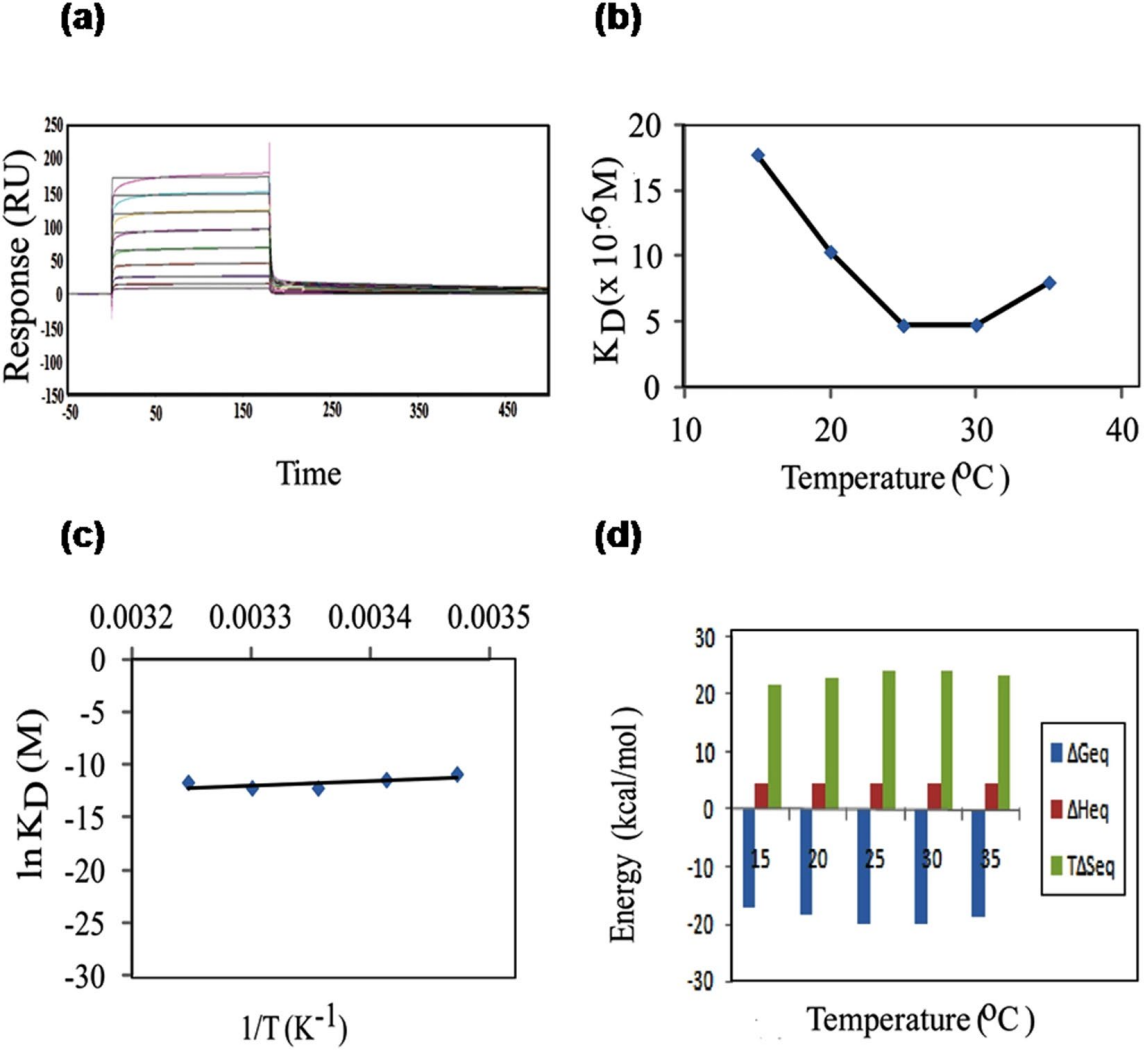
Affinity and thermodynamics for trypsin analysed through SPR. SPR based thermodynamic analysis of MP-4-trypsin interaction at equilibrium. **(a)** Typical sensorgram for MP-4 and trypsin interaction at various concentrations. **(b)** K_D_ versus temperature. **(c)** Natural log of K_D_ versus inverse of temperature, slope indicate the Arrhenius values. **(d)** Individual contributions of enthalpy (ΔH) and entropy (TΔS) to equilibrium free energy of binding (ΔG), at temperatures ranging from 15° to 35 °C.

Having established the role of temperature and pH on stability of MP-4, kinetics and thermodynamics were studied to analyse optimum binding between MP-4 and trypsin. Binding experiments were carried out on CM5 SPR chip from 15 °C to 35 °C at an interval of 5 °C. Kinetics rate constants and change in free energies at equilibrium were calculated at the same temperature ranges. A typical sensorgram is shown in Fig. 2a results from 1:1 two-state fitted models are tabulated in Table 1 and shown in Fig. 2b. At 25 °C, MP-4 binds to trypsin with k_ass_ 13.8 × 10^4^ M^−1^s^−1^ and k_diss_ 65.3 × 10^−2^ s^−1^ resulting into 4.73 μM affinity with negligible error in the fitting as reflected through sensorgram and χ^2^. It was observed that upon increase of temperature from 15 °C to 35 °C, there was a 4-fold increase in the association rate constant and approximately 2-fold increase in k_diss_ that resulted into 0.45-fold decrease in equilibrium dissociation constant (K_D_) for trypsin (Table 1).

**Table 1:** Kinetics parameters for MP-4 and trypsin binding.

Temp (°C)	k_ass_(×10^4^ M^−1^s^−1^)	k_diss_ (×10^−3^ s^−1^)	K_D_(×10^−9^ M)	R_max_ (RU)	χ^2^
15	2.76	490.4	17768.12	409.5	10.5
20	5.27	545.2	10345.35	327.8	9.26
**25**	**13.8**	**652.5**	**4728.261**	**239.9**	**7.54**
**30**	**15.4**	**735.3**	**4774.675**	**248.4**	**8.33**
35	11.1	889.5	8013.514	263	10.4

The temperature sensitivity of MP-4 binding for trypsin was further examined. Towards this purpose, enthalpy and entropy contributions for free energy of association and dissociation phase as well as the free energy at equilibrium were calculated through SPR experiments. The increase in temperature from 15 °C to 35 °C was accompanied by a decrease in K_D_ from 17.76 μM to 8.01 μM for trypsin. Data also indicate that increase in k_ass_ and k_diss_ are mainly due to increase in temperature. K_D_ versus temperature and natural log of K_D_ versus inverse of temperature for trypsin is shown in Fig. 2c, and values are tabulated in Supplementary Table S1. These values were further used in calculating the change in enthalpy (∆H), entropy (∆S) and Gibbs free energy (T∆S) during association and dissociation phases (Supplementary Table S1). The relative contributions of these modules signify the potential modes of binding. Supplementary Table S2 illustrate the change in Gibbs free energy of equilibrium upon binding of trypsin as a function of temperature. Change in enthalpy and entropy assistance towards Gibbs free energy for association and dissociation phase as well as equilibrium was estimated through Arrhenius value (natural log of K_D_ versus inverse of temperature) (Fig. 2c and d). The highest binding at equilibrium was seen at 25° and 30 °C (ΔGeq −20 Kcal/mol) while at 15 °C, 20 °C and 35 °C, it was −17, −18 and −19 Kcal/mol, respectively. Data suggest that change in enthalpy (ΔH) for trypsin from 15 °C to 35 °C is 4 Kcal/mol which is less than the reported value of 17 Kcal/mol^19^. This indicates the absence of steric hindrance at the interaction sites. While the change in entropy (T∆S) in a range of temperatures is not very significant and is positive for this complex, it is possible that solvent molecules at the interface were squeezed out during complex formation (Supplementary Table S2). It is remarkable to note that, at all temperatures, entropy is highly favourable more than compensating for marginally unfavourable enthalpy. This suggests that binding can primarily be driven by hydrophobic interactions involving the nonpolar groups.

### Docking and molecular dynamics simulation

Results of thermodynamics experiments are in accordance with studies on bioactive molecules i.e. binding affinities of MP-4 for trypsin are high at physiological temperature (25 °C and 30 °C) while drops at lower and higher temperatures. To decipher the cause of such behaviour at the molecular level, docking experiments followed by molecular dynamics simulation and interaction studies were performed. The docked complex whose binding energy was closest to the experimental value was chosen as the best structure for subsequent analysis.

Free MP-4 (PDB id: 5DSS) and trypsin (present as a complex in PDB id: 1AVW) molecules were subjected to 200 ns explicit solvent molecular dynamics simulation and clustered to choose dominant conformational states for docking (Supplementary Fig. S1). In case of MP-4, clusters with ≥ 5% population size were screened and frames from four clusters were chosen for further screening (Supplementary Table S3). While in case of trypsin, frames from the dominant cluster was chosen for subsequent analysis (Supplementary Table S3). Of these, 7 best combinations (termed as complexes henceforth) were fed into HADDOCK server for docking (Supplementary Table S4). Successfully docked complexes were analyzed based on Z-score and HADDOCK score. Surface area and binding energies of the complexes were calculated through PISA server. The best energy values obtained were −11.7 Kcal/mol and −11.4 Kcal/mol (Supplementary Table S4). Interaction studies performed on the best MP-4-trypsin model showed that only one residue (Arg71) of RSL of MP-4 was able to make electrostatic interaction with three residues (Phe39, Ser192 and Gly190) of trypsin active site. There was also limited hydrophobic interaction between Leu70, Ile69 and Phe75 of MP-4 with trypsin molecule (Phe91, Gly93, Leu96, Gly209, and Met101) in the complex.

We had also docked MP-4 with trypsin by taking into account flexibility essential for interaction in rationally designed experiment in HADDOCK server. Based on past studies, residues 187–192, 207–210 and 218–221 of trypsin and residues 67 to 72 of RSL of MP-4 were kept fully flexible as these comprised of the catalytic pocket^20^. Initially ~200 MP-4-trypsin energy minimized structures were obtained by optimized run. The top selected model had ΔG of −17.56 Kcal/mol, which is closer to the experimental value (−20 Kcal/mol at 25 °C) (Supplementary Table S5). Interaction studies of this complex showed many electrostatic and hydrophobic contacts at the interface (Supplementary Table S6). Since both interaction studies and binding energy of this docked complex yielded better results (Supplementary Table S5), we proceeded with this model for further analysis as the values justify its selection as the best model.

**Figure 3:**
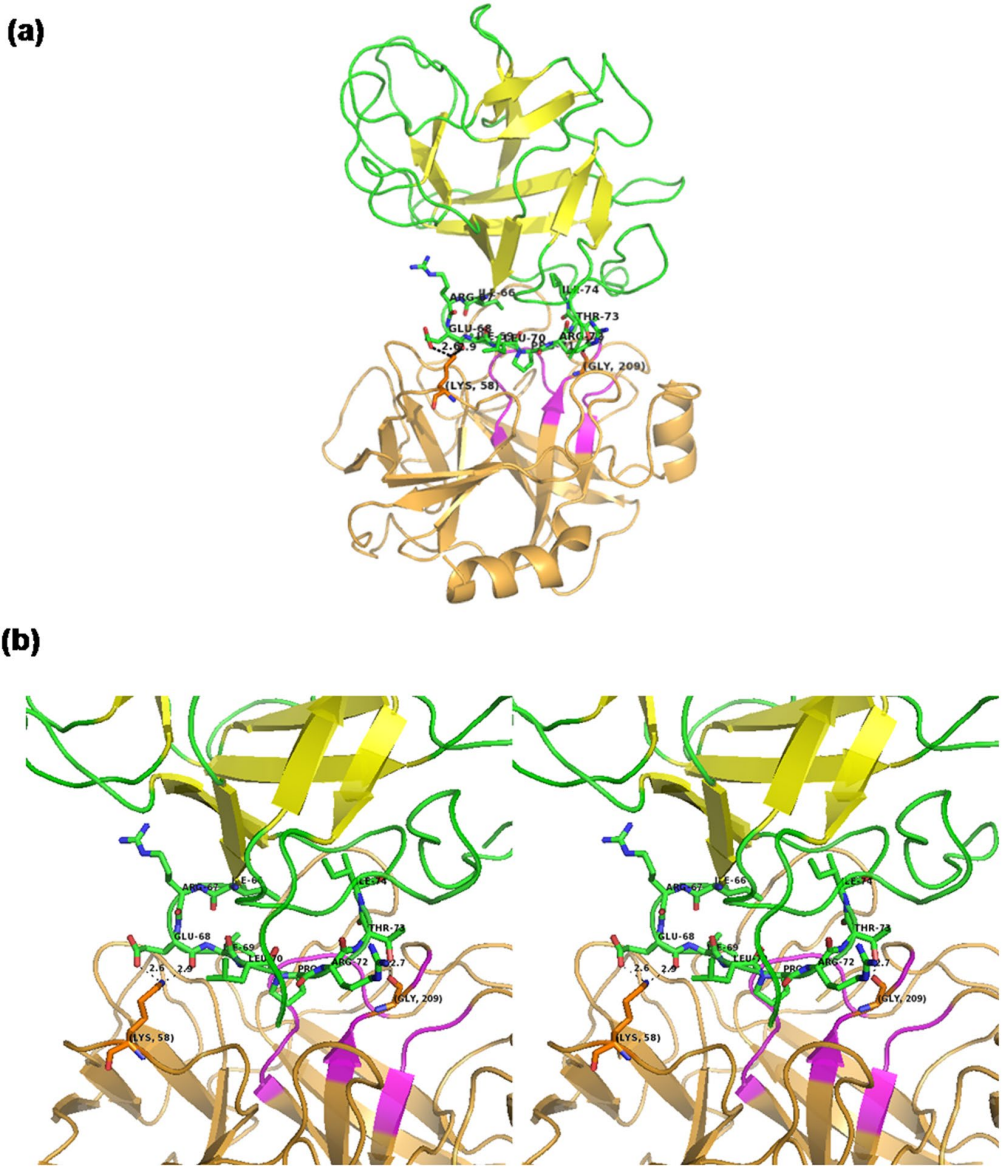
Model of MP-4-trypsin complex after docking. (**a)** Best docked structure of MP-4 (green and yellow) with trypsin (orange and magenta). **(b)** For clarity only RSL of MP-4 (green) with trypsin (orange) is shown. Magenta color of trypsin shows major dominant residues of catalytic site involve in interaction in other serine proteases. MP-4 RSL (66–74) shows limited electrostatic interaction in the catalytic pocket of trypsin. Electrostatic interactions are depicted as dotted lines.

The best docked structure of MP-4 and trypsin is shown in Fig. 3a and electrostatic interactions are shown in Fig. 3b. The analysis showed that only two residues Gln68 and Thr73 of RSL are involved in electrostatic interactions while other two residues of MP-4 Asp77 and Thr78 interact with the non-catalytic pocket of trypsin. Four residues Ile66, Ile69, Pro71 and Thr73 showed hydrophobic interactions and are not favoured by charged catalytic pocket. These interactions are further compared with known strong trypsin inhibitor complexes such as porcine pancreatic trypsin/soybean trypsin inhibitor and trypsin with bovine pancreatic trypsin inhibitor. Intensive interactions are made by the P1 residue of RSL of these two complex structures i.e. arginine and lysine. While in the case of MP-4, P1 position is occupied by isoleucine. The interaction details for MP-4 RSL with trypsin are tabulated in Supplementary Table S5. It is interesting that strong inhibitors as mentioned above show high affinity with K_D_ values 4.8 × 10^−10^ M and 6.08 × 10^−14^ M as compared to 4.7 × 10^−6^ M in MP-4-trypsin complex. This is because of deeply buried nature of P1 residues in porcine pancreatic trypsin/soybean trypsin inhibitor and trypsin with bovine pancreatic trypsin inhibitor.

The crystal structure of MP-4 RSL has been seen to be docked over catalytic part of trypsin and occupies 1029.5 Å^2^ intermolecular buried area with trypsin. In strong trypsin inhibitor complex, porcine pancreatic trypsin/soybean trypsin inhibitor (1AVW) and trypsin with bovine pancreatic trypsin inhibitor (4Y0Y) structures intermolecular buried area, 870.3 Å^2^ and 727.9 Å^2^ respectively, was calculated through PISA with extensive and effective interactions of P1 with the catalytic pocket of trypsin. In strong inhibitors, typical range of buried surface area of 600–900 Å^2^ is commonly observed^21^. Structures of RSL of MP-4, 1AVW and 4Y0Y with trypsin are shown in Supplementary Figure S2. These analyses clearly suggest that although there is a high intermolecular surface area in MP-4 trypsin complex, only limited interactions are present. This essentially implies weak inhibitory nature of this protease inhibitor. Thermodynamics data suggest that despite being a weak inhibitor, the behaviour of MP-4 can be explained to be similar to other enzymes at optimized temperature deciphered from biochemical and SPR experiments.

### Conformational/interaction features of MP-4-trypsin complex revealed through MD simulation

**Figure 4:**
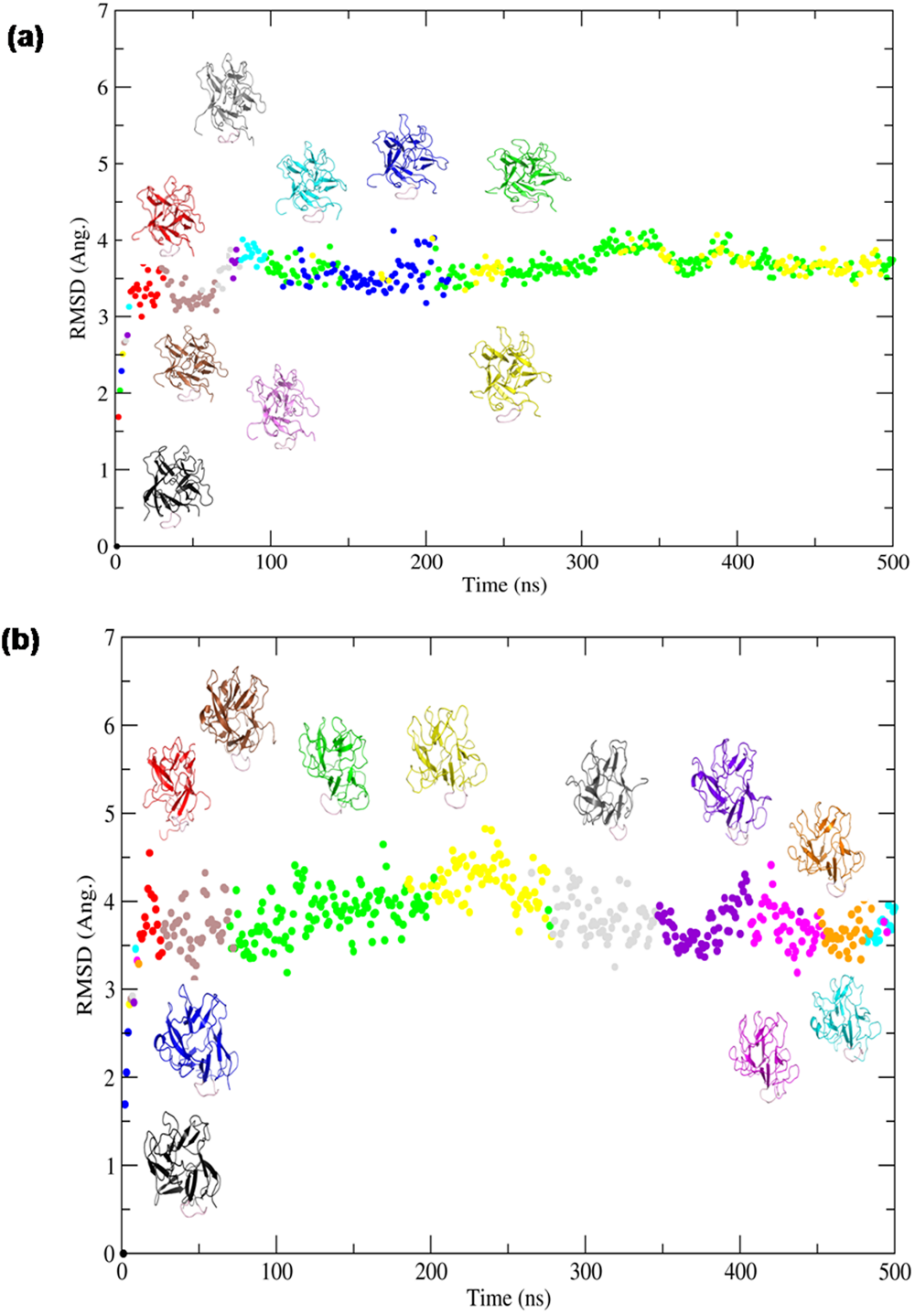
Conformational clusters of MP-4-trypsin complex. RMSD versus time plot obtained from simulation of MP-4-trypsin complex at **(a)** 15 °C and **(b)** 25 °C. Conformational clusters are color coded and least RMSD representative model of each cluster is shown along the curve.

**Figure 5:**
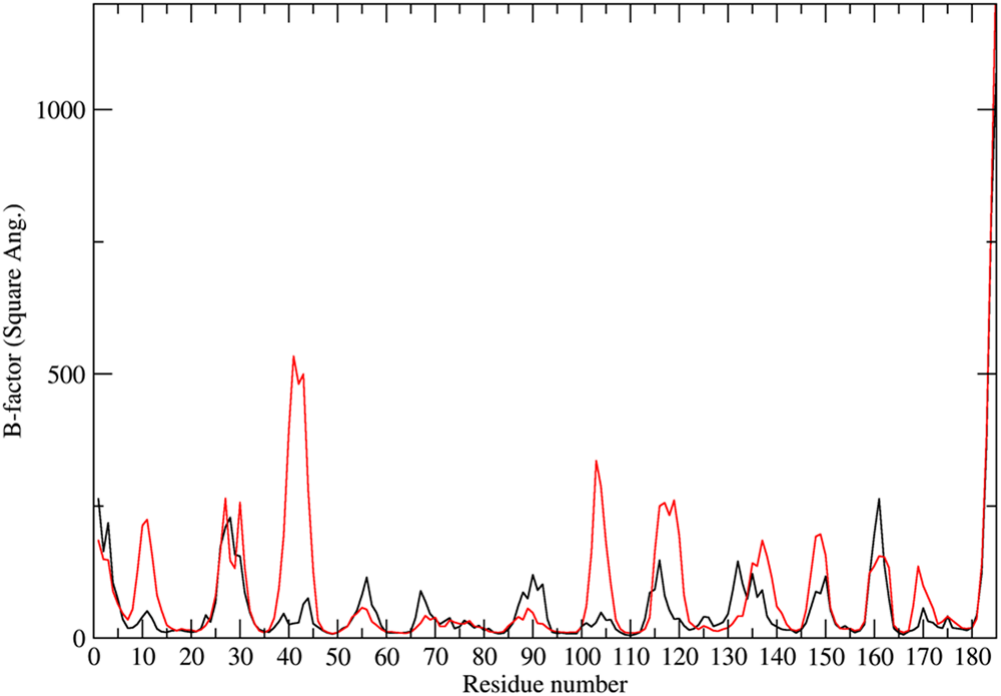
Thermal mobility of MP-4-trypsin complex. Residue-wise B-factor plot obtained from simulation of MP-4-trypsin complex at 15 °C (black) and 25 °C (red) showing flexibility of the molecule at two temperature conditions.

In order to evaluate the activity as a function of temperature, dynamics of the complex was further examined by subjecting to 500 ns molecular dynamics simulation at two different temperature conditions (15 °C and 25 °C). Conformational ensembles obtained from 15 °C and 25 °C runs were extracted and analysed using CPPTRAJ^22^ programme. The trajectory of MP-4-trypsin complex at 15 °C was stable at an RMSD of 3.0Å–4.0 Å and at 25 °C at 3.5Å-4.5 Å (Fig. 4). Frames from both runs were clustered with a radius of 2 Å of Cα from the centroid using kclust utility of MMTSB^23^ toolkit in AMBER14^24^. The number of clusters indicates inherent flexibility in the molecule to adopt different conformational states permissive for optimal binding. Nine conformational clusters were obtained for simulations at 15 °C (Fig. 4a) and 11 conformational clusters were obtained during simulation at 25 °C (Fig. 4b) indicating wider conformational landscape of a molecule for interaction at higher temperature. This was also reflected in the B-factor analysis as residue mobility was high at 25 °C as compared to that at 15 °C (Fig. 5).

**Figure 6:**
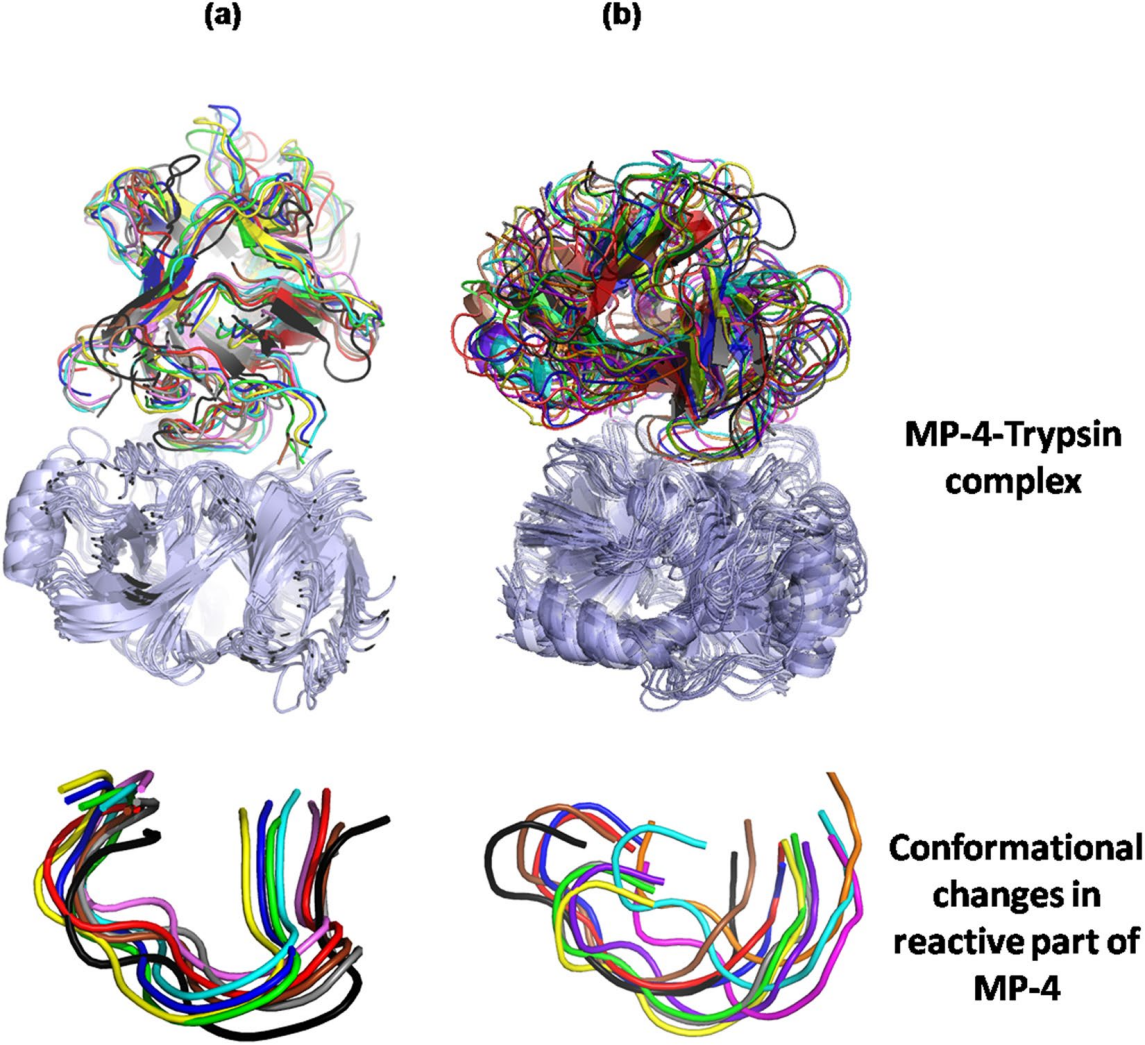
Mobility of RSL across the trajectory. Cartoon representation of structure superposition of representative frames of each cluster obtained from **(a)** 15 °C and **(b)** 25 °C simulations. Superposition of RSL from both simulations are shown in the lower panel.

Individual representative structures of each cluster were analyzed for binding energy, interface surface area, nature of interactions. At 15 °C the binding energy was in the range of −4 to −8.9 Kcal/mol with interface surface area in the range of 653 to 900 Å^2^. Due to less interface surface area, very limited electrostatic interactions and hydrophobic interactions were seen. On the contrary, at 25 °C binding energies were in the range of −2.6 to −9.6 Kcal/mol with surface area 900 to 1100 Å^2^. MP-4 and trypsin interface at 25 °C showed intense hydrogen and salt bridges along with hydrophobic interactions. The conformation of RSL was further analyzed which showed significant difference in each cluster. Interestingly, the RSL is relatively more rigid at 15 °C (Fig. 6a) in contrast to that at 25 °C (Fig. 6b). This is also reflected in number of interactions of RSL of MP-4 with trypsin molecule. At 25 °C, most of the interactions of RSL are achieved through Arg72, Ile69, Leu70, Pro71, Arg67, Thr73, Gln68, and Arg67 while at 15 °C interactions are limited to Arg67, Leu70, Arg72, and Ile74. Therefore, the RSL across the trajectory at 15 °C exhibited geometrical restrictions. The conformational window, however, was broad at 25 °C suggesting that the molecule searches all possible conformational space and different bonding patterns to find an optimal geometry for interaction at that temperature (Fig. 6b).

**Figure 7:**
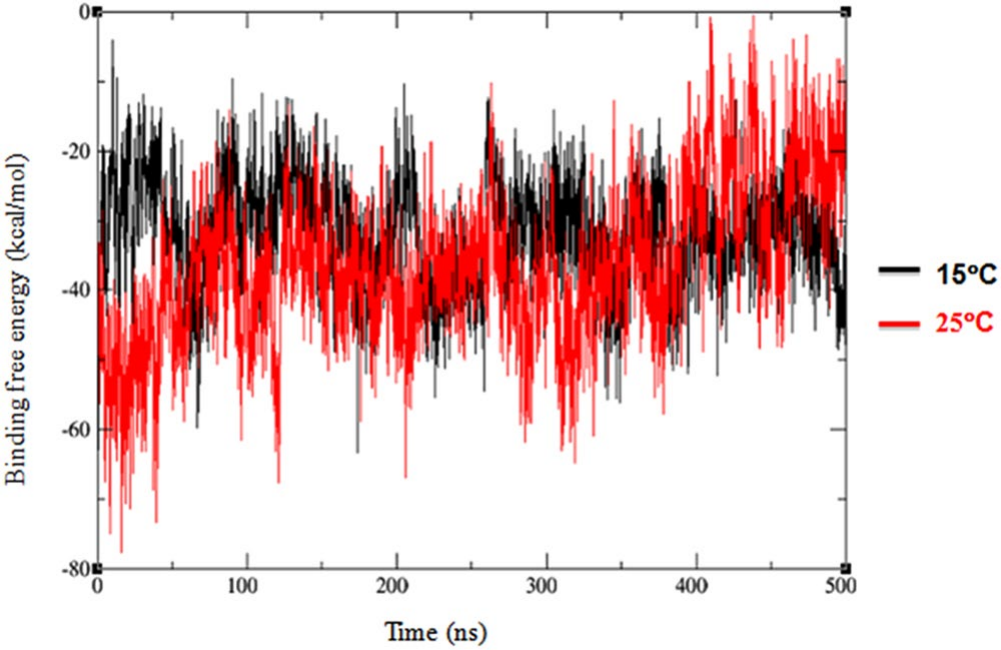
Binding free energy of MP-4-trypsin complex. Plot of free energy of binding as a measure of ∆G values across the trajectory of MP-4 trypsin complex at 15 °C (−31 Kcal/mol; black) and at 25 °C (−36 Kcal/mol; red) simulations.

Free energy of binding between MP-4 and trypsin was calculated for both the trajectories by using MMGB/SA. The free energy (ΔG) of binding across the trajectory at 15 °C was −31 Kcal/mol whereas at 25 °C it was −36 Kcal/mol (Fig. 7). These values are comparable with experimental values as ΔG was low at 25 °C as compared to that at 15 °C suggesting higher affinity at an optimum temperature similar to other receptor-ligand complexes. However, due to different sets of interactions of RSL at 25 °C, a stable complex could not be formed. Hence, MD simulation validates the weak inhibitory nature of MP-4.

## Discussion

Kunitz-type protease inhibitors (KTPI) are extremely stable molecules. Although not a classical KTPI, our study shows MP-4 to be stable even at pH 10 and temperature of 60 °C. Structure of MP-4^18^, showed that MP-4 adapts the trefoil fold similar to other KTPI. The MP-4 structure consisted of 12 antiparallel strands connected by long loops and two internal disulfide bonds between Cys45–Cys90 and Cys145–Cys152 residues. Studies suggest that the presence of intra-molecular disulfide bonds^25^, hydrophobic core and buried polar groups to be the probable reasons for their stability which is very reasonable for MP-4 structure as well^18^. Besides, like other Kunitz-type protease inhibitors, interaction of MP-4 with its cognate proteases, is governed by a positive enthalpy. Thermodynamics analysis of MP-4 has shown that enthalpy (∆H) is positive i.e. 4 Kcal/mol and ranges from 15°–35 °C.

Rise in temperature is expected to enhance the conformational mobility of loop regions in proteins. Our approach of combining molecular dynamics simulations with thermodynamic experiments shows that the behaviour of MP-4 as a function of temperature follows the conventional rule. Typically, it means that at low temperature the spectrum of accessible conformations is limited while at higher temperature it becomes flexible disabling effective interactions. Only at an intermediate temperature, the molecule is able to fine-tune its microenvironment to bind incoming ligands. Kinetics and energetics studies performed for MP-4 with trypsin enzyme in the range of 15°–35 °C show optimal binding at 25°–30 °C that decreases at low and high temperatures, as expected^26^. The data shows that conformational flexibility is limited at low temperature and the molecule tends to be in a relatively frozen state. However, an ambient temperature (25 °C in this case) facilitates thermal motion in the molecule allowing it to search the conformational space for preferential binding thereby increasing its binding affinity comparable with thermodynamics results.

Consistent with previous reports on serine protease inhibitor complexes, majority of the interacting residues are confined to the RSL of MP-4 and it has the same kind of surface protrusions that facilitate blockage of enzyme activity^27,28^. However, even at the optimal temperature of 25 °C, a stable interaction could not be achieved due to increasingly changing bonding pattern of MP-4 with trypsin across the trajectory. This could be a probable reason for MP-4 behaving as a weak inhibitor even at the optimal temperature. Apart from this, sequence variability of RSL is observed between other STI (SPYRIRFI) and MP-4 (IREILPRTI). As a consequence, MP-4 RSL does not make many hydrophilic and hydrophobic interactions in the complex vis-à-vis other STI complexes. Limited RSL intra-chain interactions as observed in most KTPIs are, however, significantly less in case of MP-4 due to which it cannot be held in the right conformation for inhibitory activity. Hence, network of interactions critical for higher affinity and inhibitory activity of protease inhibitors is absent in MP-4. The interactions formed between MP-4 and trypsin are such that the enzyme dissociates with ease and can bind to the cognate substrate to achieve proteolysis.

The effect of temperature on conformational flexibility of functionally important regions is difficult to measure utilizing biochemical or biophysical tools. The combination of thermodynamics and MD simulations employed in the present study represents a viable approach to ascertain the temperature-dependent conformational variability of functionally important regions. The MP-4 proteins are a weak protease inhibitor even though it exhibits high structural homology to KTPIs. The rationally designed experiments using a combination of thermodynamics and further validation by MD simulation has helped decipher how a protease inhibitor with coherent biochemical and thermodynamics properties with other protease inhibitors is still a weak inhibitor.

Our study validates kinetics of binding reported for various proteins in general and enzymes in particular. However, such an observation has not been reported for MP-4-trypsin complex and not even for well-known STI-trypsin complex. We have for the first time demonstrated kinetics associated with dynamics of MP-4-trypsin using different physical parameters. Coupling of *in-vitro* and *in-silico* experiments have provided new insights regarding optimal binding in this system. The same principles and diverse biophysical techniques can be applied for other protein-protein interaction behavioural studies to assess factors associated with optimal physiological interaction in a more realistic scenario. The favourable conformation of ligand is required for efficient binding with the receptor and the active conformation may be dependent on physical factors such as temperature. The rise of new function in proteins may be due to mutations that change the temperature dependent flexibility of functional regions. Drug resistance is a major public health problem of global relevance. We believe that engineering flexibility in small molecule inhibitors using the aforementioned approach will enhance conformational adaptability and prevent loss of affinity due to mutations in target protein.

## Methods

### Purification

*Mucuna pruriens* seeds were purchased from M/S Shidh Seeds Sales Corp. (Dehradun district, India). The partial characterization of seed proteome and purification of one of the dominant protein was previously standardized and reported by Kumar *et al*.^18^. Briefly, initial purification was done using pH (50 mM sodium acetate buffer, pH 5.0) based protein extraction. Gel filtration chromatography (GFC) of 60% ammonium sulfate was carried out using Sephacryl 200 preparative column (Amersham Pharmacia Biotech Inc) in 50 mM phosphate buffer, pH 7.2, with 140 mM NaCl, at a flow rate of 1 ml/min at 280 nm. The last peak on the GFC chromatogram was identified as MP-4 protein on the basis of retention time (4.5 hrs) on the GFC column (85 cm Bed height, GE Healthcare) and homogeneity was evaluated on 12% SDS-PAGE. Protein concentration was estimated by a BCA protein assay (Pierce Biotechnology) using BSA (Sigma) as the standard.

### Stability studies at various physiological conditions

Circular dichroism (CD) was performed on Jasco-700 spectropolarimeter equipped with Jasco PTC-348W temperature controller in the far-UV region (200–250 nm). 24 μM of protein was dissolved in 20 mM phosphate buffer, pH 7.2. Spectra were recorded at the temperature ranging from 20 °C–100 °C at an interval of 20 °C in a 1 mm path length quartz cuvette (200 μl, Hellma). The scanning speed was 20 nm min^−1^ and each spectrum was recorded as an average of 5 scans. The effect of pH on the secondary structure was further analysed by performing the experiment with the range of suitable buffers (citrate 3–6, phosphate 7–8 and glycine 9–10 pH range) with the same protein concentration at 37 °C. Spectra (CD mdeg) were converted into mean residue molar ellipticity [θ] MRW (deg cm^2^ dmol^−1^) and analysed using JASCO spectral analysis program.

### Preparation of the SPR sensor chip

All affinity measurements were carried out on BIAcoreT200 system (GE healthcare). 2 µM MP-4 (ligand) was dissolved in 10 mM sodium acetate pH 4.0 and amine coupled to a CM5 (carboxymethylated)-certifiedgrade sensor chips using an equal mixture of EDC/NHS (N-ethyl-N-(dimethylaminopropyl)carbodiimide; N-hydroxysuccinimide) (BIAcore amine coupling kit). Approximately 178 RU was achieved on immobilization and such less immobilization ensured the minimization of mass transport effect. The protein was immobilized at the flow rate of 5 μl min^−1^ for 120 seconds and the unreacted active sites were blocked with 1 M ethanolamine (BIAcore amine coupling kit). The running buffer was composed of 10 mM HEPES pH 7.4 containing 150 mM NaCl, 3 mM EDTA and 0.005% surfactant P20 (GE Healthcare). One flow cell was not immobilized with protein whereas EDC/NHS and ethanolamine was used. This flow cell treated as a control for monitoring any non-specific binding of ligand.

### Acquisition of kinetic binding data

The binding experiments were performed in the range of 10 °C–35 °C temperature at an interval of 5 °C. Various concentrations of analyte i.e. for trypsin 32 µM-125 nM were injected into flow cell at a particular temperature. Each cycle comprised of 3 min association phase and 15 min dissociation phase. Regeneration of chip was achieved by 0.1 N NaCl. Kinetics data were analysed using BIAcore T200 Evaluation Software version.1.0. Model was fitted using 1:1 Langmuir binding interaction model system describing 1:1 binding between analyte (A) and ligand (L) [A + L ↔ AL] as well as we have also seen data are more appropriately fitting in two-state (conformational change) model [A + L ↔ AL ↔ AL*]^29–31^. Equilibrium dissociation constant (K_D_ = kd/ka) was calculated from fitted sensorgram. Chi-square (χ^2^) value was strictly monitored during the data fitting. χ^2^ is the standard statistical value indicates signal-to-noise ratio or the closeness of the fit between the experimental data and the model. Typically, χ^2^ values should be less than 10 percent of Rmax.

### Calculation of thermodynamics parameters

Equilibrium dissociation constant (K_D_) values for each set of experiments were further used for calculation of free energies of reaction between analyte and ligand. ∆Geq = −RTlnK_D_/c_o_, R is the Rydberg’s constant (1.09677 × 10^3^ m^−1^), T is the temperature in Kelvin, and c_o_ is the standard state concentration (1 mol l^−1^). Heq was derived by directly fitting the experimental data to the integrated form of van’t Hoff equation K_D_ = K°_D_ e^H°/R(1/T°−1/T)^e^CT°/R(1/T°−1/T)^ (T°/T)^C/R^, where C refers to heat capacity change at constant pressure and the degree symbol refers to parameter values at a reference temperature. Entropy change was then calculated as *∆Geq = ∆Heq−T∆Seq*.

### Molecular Docking

HADDOCK web server v.2 is a robust global docking program based on molecular mechanics (MM) and performs rigid body energy minimization, simulated annealing and water refinement^32^. The program searches conformational space of the two protein partner in the system and docks the molecules in low-energy orientations by either involve all-atom structures or through some degree of coarse-graining. The advantage of HADDOCK is that one can explicitly define the backbone flexibility hence provide conformational plasticity while retaining the biochemical information.

MP-4 structure (PDB id-5DSS) was docked to trypsin *in-silico* by using HADDOCK web server. MP-4 structure was aligned to soybean trypsin inhibitor (STI) structure in soybean trypsin inhibitor-trypsin complex (PDB id-1AVW). The coordinates of STI were substituted by the coordinates of MP-4 in the complex. In the defined docking mode, residues 187–192, 207–210 and 218–221 of trypsin were kept as active residues because these stretches of amino acids are generally involved in interaction in the other known complexes. Similarly, residues from 67 to 72 of MP-4 were defined as active flexible residues. Approximately 200 structures in 5 clusters were obtained from HADDOCK server, which represented 95% of the water-refined models. The top cluster of MP-4-trypsin was selected primarily on the basis of Z-score and change in free energy, Kcal/mol (∆G). PISA server (http://www.ebi.ac.uk/msd-srv/prot_int/) was used to calculate total buried surface area, nature of interactions and residues involved in interactions at docking site^33^. Visualization and preparation of structure figures were done using PyMOL^34^.

### Molecular Dynamics (MD) simulation

The best model of MP-4-trypsin complex obtained from HADDOCK server was assessed for various parameters and the complex was manually analysed for steric clashes with neighbouring residues. PROCHECK^35^ was used for analysing the stereochemistry of the residues. On the basis of minimal clash and better values of Z-score, ∆G and stereochemistry, the best model was selected for molecular dynamics simulation.

Molecular Dynamics Simulations were carried out for free MP-4 and trypsin for 200 ns and for the selected best docked model for 500 ns at 15 °C and 25 °C using ff12SB force field of Amber 14^24,36,37^. The structures were explicitly solvated with a 10 Å TIP3P water box from the outermost atoms of the molecules. Periodic boundary conditions were used and the net negative charge neutralized with Na^+^ counterion using tleap program of AMBER 14^38^. Prior to production dynamics; temperature and pressure were equilibrated at 25 °C and 15 °C and at 1 atmospheric pressure. At constant volume, equilibration was performed for 50 ns. The coordinates were saved after every 10 ps during the simulation such that the 50 ns trajectories consisted of 5000 MD sub-structures. All simulations were carried out with sander and performed on CUDA version of PMEMD NVIDIA K20X GPU support from Amber 14 accessible from RHEL workstation. All simulations were repeated to verify AMBER results.

### Analysis of MD Trajectories

Trajectories were analysed using CPPTRAJ program. 500 intermediates/snapshots were extracted out from the 500 ns AMBER trajectories at an interval of 1 ns. The extracted frames were clustered for comparative analyses of conformational ensembles obtained from 15 °C and 25 °C simulations. Clustering was performed by kclust utility in the MMTSB (Multiscale Modeling Tools for Structural Biology) suite with clustering radius of 2.0 Å from the centroid for backbone, Cα^23^. Various types of structural properties were evaluated by calculating the backbone root-mean-square deviation (RMSD), H-bond analysis. Investigation of mobility in the molecules at the two temperatures were done by estimation of flexibilities of residues in terms of root-mean-square fluctuation (RMSF) and B-factors using CPPTRAJ module of AMBER14 for frames extracted at 100 ps. Molecular Mechanics Generalized Born Surface Area (MM-GBSA)^39^ was used to measure binding energy (∆G) between the complexes at 25 °C and 15 °C. The 5000 snapshots obtained throughout the 500 ns simulations were applied for MM-GBSA free energy calculations. The method has been used as a reliable technique for accurate calculation of free energy of binding (∆Gbind) using the following equation:

$${\rm{\Delta }}Gbind={\rm{\Delta }}Gcomplex-{\rm{\Delta }}Greceptor-{\rm{\Delta }}Gligand$$

## Electronic supplementary material

Supplementary data
