# Supplementary material for: Docking, thermodynamics and molecular dynamics (MD) studies of a non-canonical protease inhibitor, MP-4, from Mucuna pruriens
Source: Sci Rep. 2018 Jan 12;8:689. doi: 10.1038/s41598-017-18733-9 (PMC5766534; doi:10.1038/s41598-017-18733-9)
Supplement: Supplementary file 1 — Supplementary data [file 41598_2017_18733_MOESM1_ESM.doc]

**Docking, thermodynamics and molecular simulation (MD) studies of a non-**

**canonical protease inhibitor, MP-4, from *Mucuna pruriens***

**Ashish Kumar^1^,**^§,¶^**, Harmeet Kaur^1,2^,**^§^**, Abha Jain^1^, Deepak T. Nair**^1^ **and Dinakar M.**

**Salunke**^1, 3*^

**^1^**Regional Centre for Biotechnology, NCR Biotech Science Cluster, 3^rd^ Milestone, Faridabad-Gurgaon Expressway, Faridabad - 121001 India

^2^Manipal University, Manipal, 576104, Karnataka, India

^3^International Centre for Genetic Engineering and Biotechnology (ICGEB), Aruna Asaf Ali

Marg, New Delhi 110067 India.

**Supplementary Data**

---------------

^§^Both authors contributed equally to this work.

^¶^Current address: The Pennsylvania State University, 469 North Frear, University Park, PA 16802, USA

**^*^**To whom correspondence should be addressed:

Dinakar M. Salunke

International Centre for Genetic Engineering and Biotechnology (ICGEB),

Aruna Asaf Ali Marg, 110067

New Delhi, India.

Telephone number: +91-11-26742317;

Fax number: +91-11-26742316;

E-mail: dinakar.salunke55@gmail.com

**Supplementary Figure S1. RMSD versus time plot of free MP-4 and free trypsin**

RMSD plot of the trajectories obtained from 200ns simulation of (A) free MP-4 and (B) free trypsin molecules. Average RMSD of free MP-4 across 200ns was in the range of 4-4.5Å while that of free trypsin was 1.5Å.


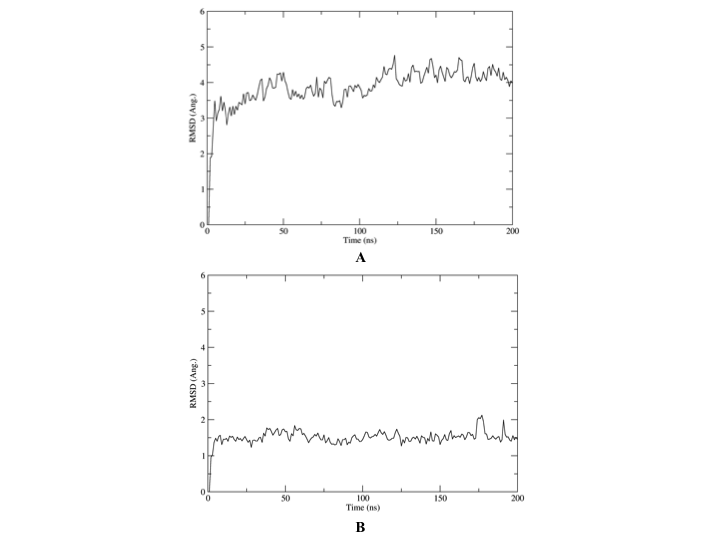


**Supplementary Figure S2. Visual depiction of P1 of RSL in strong protease inhibitors and MP-4 with trypsin**

MP-4 trypsin best docked structure, porcine pancreatic trypsin/soybean trypsin inhibitor (PDB id: 1AVW) and trypsin with bovine pancreatic trypsin inhibitor (PDB id: 4Y0Y). Trypsin is shown in wheat, yellow and pale green color and RSL are in orange, magenta and salmon color in these complex structures respectively.


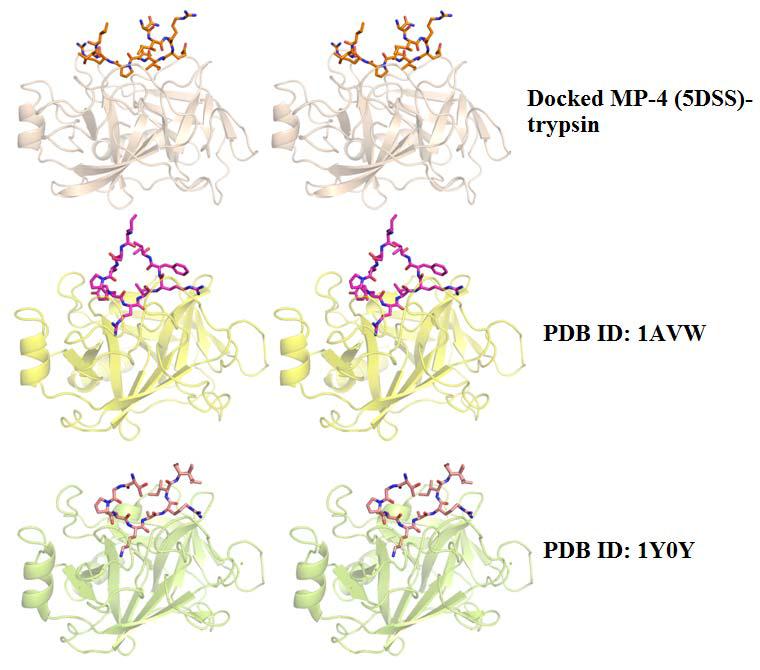


**Supplementary Table S1: Thermodynamic values for MP-4 and trypsin**

|  |  |  | **Eyring value** | | **Free energy** | | |  | **Enthalpy** |  |  | **Free** |
| --- | --- | --- | --- | --- | --- | --- | --- | --- | --- | --- | --- | --- |
|  |  |  | [Rln(Kah/kBT)] | | (∆G) | |  |  | (∆H) |  |  | **energy**(T∆S) |
| **Van't Hoff** | |  |  |  | (kcal/mol) | |  |  | (kcal/mol) |  |  | (kcal/mol) |
| 1/T (K) |  | ln KD | Ass | diss | Ass |  | diss | Eq | ass | Diss | eq | eq |
|  |  |  |  |  |  |  |  |  |  |  |  |  |
| 0.003472 |  | -10.94 | -38.14 | -59.88 | -531.83 |  | -514.46 | -17.37 | -571.96 | -576.34 | 4.38 | 21.748 |
|  |  |  |  |  |  |  |  |  |  |  |  |  |
| 0.003413 |  | -11.48 | -36.893 | -59.70 | -543.016 |  | -524.57 | -18.44 | -581.896 | -586.28 | 4.38 | 22.822 |
|  |  |  |  |  |  |  |  |  |  |  |  |  |
| **0.003356** |  | **-12.26** | **-35.015** | **-59.38** | **-554.83** |  | **-534.83** | **-19.998** | **-591.831** | **-596.21** | **4.38** | **24.378** |
|  |  |  |  |  |  |  |  |  |  |  |  |  |
| **0.0033** |  | **-12.25** | **-34.83** | **-59.17** | **-564.95** |  | **-544.97** | **-19.9784** | **-601.766** | **-606.15** | **4.38** | **24.358** |
|  |  |  |  |  |  |  |  |  |  |  |  |  |
| 0.003247 |  | -11.73 | -35.51 | -58.83 | -574.202 |  | -555.25 | -18.9492 | -611.701 | -616.08 | 4.38 | 23.329 |
|  |  |  |  |  |  |  |  |  |  |  |  |  |

**Supplementary Table S2: Energy calculation for MP-4 and trypsin**

| Temp |  |  | **25** | **30** |  |  |
| --- | --- | --- | --- | --- | --- | --- |
| (°C) | 15 | 20 |  |  | 35 |  |
| ∆Geq | -17 | -18 | **-20** | **-20** | -19 |  |
| ∆Heq | 4 | 4 | **4** | **4** | 4 |  |
| T∆S_eq_ | 21 | 22 | **24** | **24** | 23 |  |

**Supplementary Table S3: Percent population of conformational clusters from 200ns simulations of free MP-4 and free trypsin.**

| **MD simulation of free MP-4 (200ns)** | |
| --- | --- |
| Cluster number | % Population |
| 1 | 15 |
| 2 | 11.5 |
| 3 | 14 |
| 4 | 7 |
| 5 | 1.5 |
| 6 | 4.5 |
| 7 | 1 |
| 8 | 1.5 |
| 9 | 3 |
| 10 | 10 |
| 11 | 5.5 |
| 12 | 1.5 |
| 13 | 1 |
| 14 | 1.5 |
| 15 | 2 |
| 16 | 6.5 |
| 17 | 1.5 |
| 18 | 5 |
|  | |
| **MD simulation of free Trypsin (200ns)** | |
| Cluster number | % Population |
| 1 | 91 |
| 2 | 9 |

**Clusters of MP-4 with ≤ 0.5% population have been excluded as the population size was insignificant*.

**Supplementary Table S4: Screening and docking of the best snapshots (obtained from 200ns simulation) in HADDOCK server and interface analysis of the best poses through PISA server.**

| **No.** | **Complexes built from snapshots obtained from MD of MP4 and trypsin** | **HADDOCK Docking** | | | | | **Top model from each cluster selected for PISA analysis** | |
| --- | --- | --- | --- | --- | --- | --- | --- | --- |
|  |  | **Total structures and total cluster number** | **Cluster(s)** | **Haddock score** | **Z-score** | **Cluster size** | **Binding energy kcal/mol** | **Interface area, Å^2^** |
| 1 | Complex 1 | 165 structures in 12 cluster (s) | Cluster 3 | -62.6 +/- 2.9 | -1.2 | 24 | -7.9 | 813.2 |
|  |  |  | Cluster 2 | -59.9 +/- 3.7 | -0.8 | 40 | -6.7 | 647.3 |
|  |  |  | Cluster 1 | -49.9 +/- 3.9 | 0.5 | 44 | -10.3 | 657.2 |
| 2 | Complex 2 | 171 structures in 15 cluster(s) | Cluster 2 | -79.5 +/- 7.4 | -1.5 | 22 | -5.7 | 790.7 |
|  |  |  | Cluster 1 | -75.4 +/- 4.6 | -1.0 | 33 | -11.7 | 878.5 |
|  |  |  | Cluster 4 | -69.4 +/- 3.1 | -0.3 | 18 | -9.8 | 780.5 |
|  |  |  | Cluster 3 | -57.2 +/- 2.2 | 1.2 | 20 | -5.8 | 764.9 |
|  |  |  | Cluster 5 | -56.3 +/- 9.8 | 1.3 | 15 | -8.5 | 837.1 |
| 3 | Complex 3 | 163 structures in 12 cluster(s) | Cluster 3 | -95.7 +/- 4.4 | -1.6 | 24 | -7.3 | 1153.0 |
|  |  |  | Cluster 4 | -78.5 +/- 10.0 | -0.9 | 22 | -9.1 | 1037.1 |
|  |  |  | Cluster 1 | -71.4 +/- 3.9 | -0.6 | 33 | -7.6 | 755.6 |
|  |  |  | Cluster 2 | -60.4 +/- 7.4 | -0.1 | 26 | -8.7 | 838.6 |
| 4 | Complex 4 | 167 structures in 8 cluster(s) | Cluster 1 | -99.2 +/- 9.4 | -2.2 | 72 | -11.4 | 836.4 |
|  |  |  | Cluster 5 | -77.8 +/- 6.7 | -0.9 | 13 | - 8.5 | 831.1 |
|  |  |  | Cluster 4 | -61.3 +/- 3.9 | 0.1 | 17 | -3.5 | 767.8 |
|  |  |  | Cluster 2 | -50.1 +/- 2.8 | 0.8 | 27 | -10.2 | 596.0 |
|  |  |  | Cluster 3 | -48.9 +/- 3.2 | 0.9 | 19 | -10.3 | 727.5 |
| 5 | Complex 5 | 179 structures in 7 cluster(s) | Cluster 1 | -95.2 +/- 2.3 | -1.5 | 71 | -10.5 | 1073.4 |
|  |  |  | Cluster 4 | -90.5 +/- 6.1 | -1.2 | 15 | -10.7 | 1034.7 |
|  |  |  | Cluster 2 | -77.9 +/- 7.9 | -0.4 | 36 | -10.1 | 830.7 |
|  |  |  | Cluster 3 | -54.9 +/- 3.1 | 1 | 32 | -7.7 | 781.0 |
| 6 | Complex 6 | 194 structures in 9 cluster(s) | Cluster 1 | -85.5 +/- 5.4 | -1.3 | 105 | -10.2 | 666.2 |
|  |  |  | Cluster 2 | -84.5 +/- 5.7 | -1.2 | 35 | -10.7 | 986.2 |
|  |  |  | Cluster 3 | -77.4 +/- 5.4 | -0.8 | 14 | -9.6 | 843.8 |
|  |  |  | Cluster 4 | -48.5 +/- 5.3 | 0.8 | 13 | -10.2 | 574.1 |
| 7 | Complex 7 | 186 structures in 8 cluster(s) | Cluster 2 | -96.0 +/- 5.4 | -1.7 | 23 | -6.6 | 1067.4 |
|  |  |  | Cluster 4 | -88.7 +/- 4.7 | -1.2 | 14 | -9.7 | 889.9 |
|  |  |  | Cluster 1 | -77.3 +/- 4.0 | -0.6 | 108 | -10.5 | 683.3 |
|  |  |  | Cluster 3 | -64.4 +/- 8.0 | 0.2 | 15 | -10.6 | 785.9 |
|  |  |  | Cluster 5 | -54.3 +/- 5.2 | 0.8 | 11 | -10.7 | 827.7 |

**Supplementary Table S5: Comparison of the results from docking experiments**

| **Docking experiments** | **Docking hits** | **Screened out** | **Best** | **∆ G Value**  **(Kcal/mol)** | |
| --- | --- | --- | --- | --- | --- |
|  |  |  |  | **Predicted** | **Experimental** |
| MD followed by docking | 1225 (from 7 complexes) | 2 | 1 | -11.7 | - 20.0  (at 25°- 30°C) |
| Docking by flexibility in the catalytic site | 200 | 3 | 1 | -17.56 |  |

**Supplementary Table S6: Interaction studies of MP-4 with trypsin using docking experiment.**

(A) Electrostatic interacting residues of MP-4 and trypsin. (B) Hydrophobic interactions within 5.0Å distance between various interacting residues of MP-4 and trypsin.

**A**

|  |  | **S. No.** |  | **MP-4** |  | **Distance (Å)** | | | **Trypsin** |  |
| --- | --- | --- | --- | --- | --- | --- | --- | --- | --- | --- |
|  |  |  |  |  |  |  |  |  |  |  |
|  |  | 1 |  | A:Gln 68 [OE1] |  | 2.64 |  |  | B:Lys 58[NZ] |  |
|  |  |  |  |  |  |  |  |  |  |  |
|  |  | 2 |  | A:Gln 68[O] |  | 2.94 |  |  | B:Lys 58[NZ] |  |
|  |  |  |  |  |  |  |  |  |  |  |
|  |  | 3 |  | A:Thr 73[OG1] |  | 2.66 |  |  | B:Gly 209[O] |  |
|  |  | 4 |  | A:Asp 77[O] |  | 2.79 |  |  | B:Gly 143[N] |  |
|  |  |  |  |  |  |  |  |  |  |  |
|  |  | 5 |  | A:Thr 78[OG1] |  | 2.86 |  |  | B:Ser 144[OG] |  |
|  |  |  |  |  |  |  |  |  |  |  |
| **B** | | |  |  |  |  |  |  |  |  |
|  |  | **S. No** | **MP-4** | | **Trypsin** | |  |  |  |  |
|  |  | |  | |  | | |  |  |  |
|  | 1 | | A:Ile 66 | | B:Tyr146 | | |  |  |  |
|  | **2** | | **A:Ile 69** | | **B:Phe39, His38,** | | |  |  |  |
|  |  |  |  |  | **Gly190, Tyr146** | | |  |  |  |
|  |  |  |  |  |  |  |  |  |  |  |
|  | 3 | | A:Pro 71 | | B:Ser192, Gln189,  Trp208 | | |  |  |  |
|  |  |  |  |  |  |  |  |  |  |  |
|  |  |  |  |  |  |  |  |  |  |  |
|  | 4 | | A:Thr 73 | | B:Tyr210, | | |  |  |  |
|  |  |  |  |  | Gly209 | |  |  |  |  |
|  |  |  |  |  |  |  |  |  |  |  |
